# Quantification of Photocyanine in Human Serum by High-Performance Liquid Chromatography-Tandem Mass Spectrometry and Its Application in a Pharmacokinetic Study

**DOI:** 10.1155/2014/102474

**Published:** 2014-06-24

**Authors:** Bing-Tian Bi, Ben-Yan Zou, Li-Ting Deng, Jing Zhan, Hai Liao, Kun-Yao Feng, Su Li

**Affiliations:** ^1^State Key Laboratory of Oncology in South China, Collaborative Innovation Center of Cancer Medicine, Sun Yat-sen University Cancer Center, Guangzhou, Guangdong 510060, China; ^2^Department of Clinical Trial Center, Cancer Center, Sun Yat-sen University, Guangzhou, Guangdong 510060, China; ^3^Department of Medical Oncology, Cancer Center, Sun Yat-sen University, Guangzhou, Guangdong 510060, China

## Abstract

Photocyanine is a novel anticancer drug. Its pharmacokinetic study in cancer patients is therefore very important for choosing doses, and dosing intervals in clinical application. A rapid, selective and sensitive high-performance liquid chromatography-tandem mass spectrometry (HPLC-MS/MS) method was developed and validated for the determination of photocyanine in patient serum. Sample preparation involved one-step protein precipitation by adding methanol and N,N-dimethyl formamide to 0.1 mL serum. The detection was performed on a triple quadrupole tandem mass spectrometer operating in multiple reaction-monitoring (MRM) mode. Each sample was chromatographed within 7 min. Linear calibration curves were obtained for photocyanine at a concentration range of 20–2000 ng/mL (*r* > 0.995), with the lower limit of quantification (LLOQ) being 20 ng/mL. The intrabatch accuracy ranged from 101.98% to 107.54%, and the interbatch accuracy varied from 100.52% to 105.62%. Stability tests showed that photocyanine was stable throughout the analytical procedure. This study is the first to utilize the HPLC-MS/MS method for the pharmacokinetic study of photocyanine in six cancer patients who had received a single dose of photocyanine (0.1 mg/kg) administered intravenously.

## 1. Introduction

Photodynamic therapy (PDT) is a potential model for cancer therapy, which has been used to treat or relieve the symptoms of skin cancer, esophageal cancer, prostate cancer, and non-small cell lung cancer [1–3]. During the PDT procedure, the excited photosensitizer forms highly reactive oxygen species using visible light of an appropriate wavelength, resulting in oxidative damage to cellular membranes and membranous organelles [2, 4–7].

As one type of the photosensitizer, porphyrins have been approved for PDT in the USA, Europe, Canada, and Japan, but their weak absorption attenuates their optimal application in PDT [8]. Phthalocyanines and their derivatives are also widely used photosensitizers for the PDT of cancer, displaying high absorption of visible light, mainly in the phototherapeutic wavelength window (600–800 nm) [9, 10]. Photosense, Pc4, and CGP55847 are second-generation photosensitizers that have been used in clinical practice [11–14].

**Figure 1:**
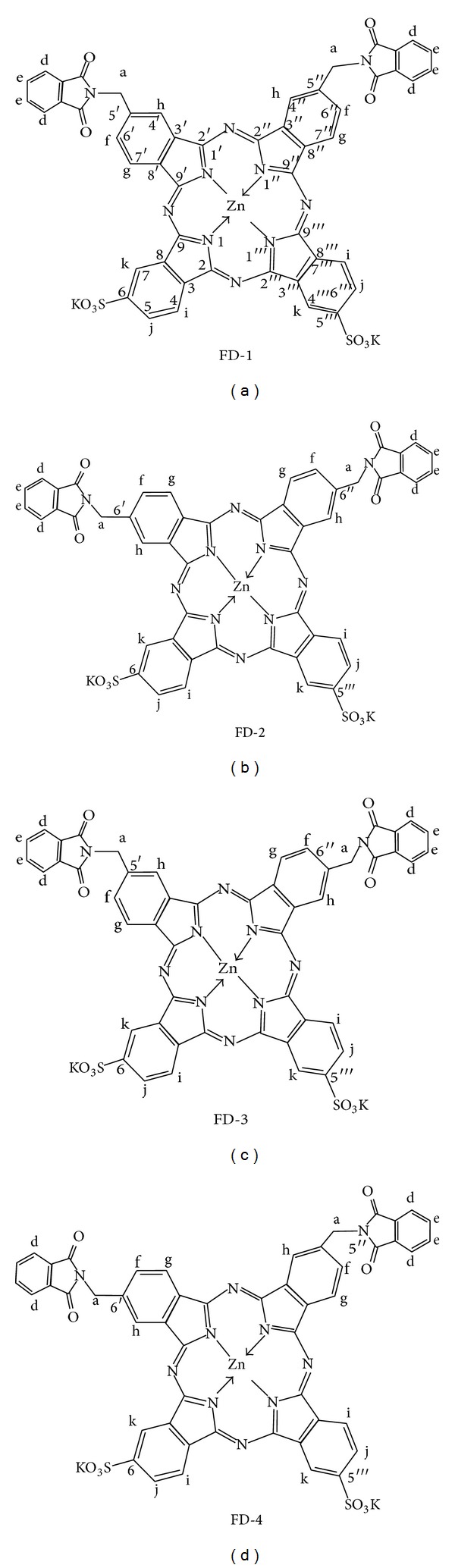
Chemical structures of photocyanine.

Photocyanine (ZnPcS2P2), a new second-generation PDT drug, was approved for clinical trials as a new medicine in 2008 by the State Food and Drug Administration in China. It is an isomeric mixture of di-(potassium sulfonate)-di-phthalimidomethyl phthalocyanine zinc (Figure 1) [15–17]. The presence of both hydrophobic and hydrophilic groups in photocyanine would improve its tumor selectivity. Clinical trial of a novel synthesized drug requires a reliable method for measuring levels of the drug in biological samples. Because photocyanine is a novel photodynamic drug, few methods have been developed for its quantification. Li et al. [18] also reported an HPLC method to separate the four isomers of a photocyanine mixture from human serum. However, the detection signals from this method display insufficient specificity in the biological samples, because it is difficult to avoid the interference from the matrix or other interferents by ultraviolet detector. Therefore, a method with higher specificity should be established to ensure the validity of the determination.

No evidence verifies the difference of each isomer of photocyanine in pharmacodynamic studies. Moreover, no standard substances for any of the isomers can be obtained from Fujian Longhua Pharmaceutical Co. Therefore, we developed a new high-performance liquid chromatography-tandem mass spectrometry (HPLC-MS/MS) method to determine the total concentration of photocyanine in cancer patient serum in a pharmacokinetic study. The method was validated for its specificity, sensitivity, linearity, accuracy, precision, matrix effect, dilution integrity, and stability, and the data established the method as a high-throughput and reliable bioanalytical assay.

## 2. Materials and Methods

### 2.1. Experimental Chemicals

Photocyanine (purity > 95%) and the internal standard (IS) mono-*β*-sulfonated zinc phthalocyanine potassium (purity > 95%) were provided by Fujian Longhua Pharmaceutical Co. (Fujian, China). HPLC-grade N, N-dimethyl formamide (DMF) and methanol were purchased from Tedia Company, Inc., (Fairfield, OH, USA). Aqueous ammonia was obtained from Guangzhou Chemical Reagent Factory (Guangzhou, Guangdong, China). Deionized water was obtained from a Milli-Q analytical deionization system (Millipore, Bedford, MA, USA). Freshly obtained, drug-free human serum was collected from healthy individuals and stored at −80°C before use.

### 2.2. Chromatographic Conditions

The HPLC system consisted of an LC-20AD solvent delivery system, an SIL-20AC autosampler, a CTO-20AC column oven, and a CBM-20A controller from Shimadzu (Kyoto, Japan). Chromatographic separation of photocyanine and mono-*β*-sulfonated zinc phthalocyanine potassium was evaluated on an XBridge C18 column (50 mm × 4.6 mm, 5 *μ*m) from Waters (Milford, MA, USA). For method validation and sample analysis, chromatographic separation was conducted by gradient elution using deionized water (adjusted to pH 10.0 with aqueous ammonia) as mobile phase A (MPA) and methanol as mobile phase B (MPB). The HPLC program for gradient elution was as follows: 20% of MPB (0–0.2 min), from 20% to 95% of MPB (0.2–1.3 min), 95% of MPB (1.3–4.0 min), from 95% to 20% of MPB (4.0–4.1 min), and 20% of MPB (4.1–7.0 min). The separation was performed using a flow rate of 0.6 mL/min. The column temperature was maintained at 60°C.

### 2.3. Mass Spectrometric Conditions

An API 4000 QTRAP system (AB SCIEX, MA, USA) was operated in negative ionization mode with multiple reaction monitoring (MRM) for HPLC-MS/MS analysis. The mass spectrometric parameters were optimized to improve the MRM sensitivity. The instrument parameters for monitoring photocyanine and IS were as follows: vaporizer temperature, 650°C; ion spray voltage, −4,500 V; curtain gas (CUR), nitrogen, 25; nebulizing gas (GS1), 65; heated gas (GS2), 65; declustering potential (DP), photocyanine −140 V, IS −135 V; collision energy (CE), photocyanine −50 eV, IS −64.4 eV; entrance potential (EP), −10 V; collision cell exit potential (CXP), −10 V. The precursor-to-product ion transitions used for the MRM of photocyanine and IS were* m/z* 526.0 → 146.0 and* m/z* 655.1 → 591.8, respectively. The mass spectrometer was operated at unit mass resolution for both the first and third quadrupoles.

### 2.4. Sample Preparation

A 100 *μ*L aliquot of blank human serum, spiked serum, or pharmacokinetic study serum was transferred to a 1.5 mL Eppendorf tube. Then, 200 *μ*L of DMF was added to each tube of serum, and the mixture was vortexed for 1 min. The mixture was then spiked with 300 *μ*L methanol containing 450 ng/mL IS, vortexed, and centrifuged for 10 min at 15,000 rpm at 4°C. The supernatant was collected and filtered. 10 *μ*L of supernatant was injected into the LC-MS/MS system for analysis.

### 2.5. Method Validation

**Figure 2:**
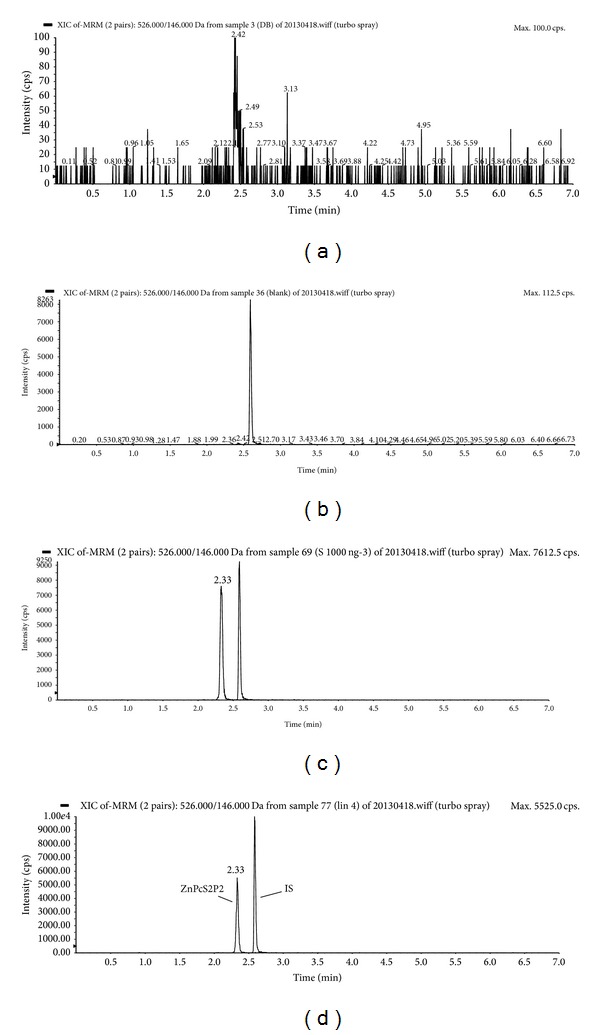
Representative HPLC-MS/MS chromatograms of extract from (a) blank serum; (b) a human serum sample spiked with mono-*β*-sulfonated zinc phthalocyanine potassium (IS), (c) a human serum sample spiked with 1000 ng/mL photocyanine and IS, and (d) a patient serum sample after i.v. administration of photocyanine spiked with IS.

Photocyanine was validated for an HPLC-MS/MS assay. Specificity, the lower limits of quantification (LLOQ), linearity, accuracy, precision, extraction recovery, matrix effect, and stability were evaluated during method validation. The specificity was assessed by testing six independent aliquots of blank serum for exclusion of any endogenous interference at the peak region of photocyanine or IS (Figure 2). LLOQ was defined as the lowest concentration on the standard calibration curve from six different batches, in which both precision and accuracy were ≤20% with a signal-to-noise ratio (S/N) > 10. The linearity of the calibration curve was evaluated over the range of 20 ng/mL and 2000 ng/mL. Calibration curves were constructed via linear least-squares regression analysis by plotting the peak-area ratios (photocyanine/IS) versus the drug concentrations in the serum, and the resulting correlation coefficient (*r* > 0.99) was considered satisfactory. Precision and accuracy were assessed by the analytes covering the range of the calibration curve, in which the criteria for acceptability are defined as an accuracy ±15% standard deviation (SD) from the nominal values and a precision of ±15% relative standard deviation (RSD). Intrabatch accuracy and precision were evaluated by analyzing the quality control (QC) samples at concentrations of 60, 1000, and 1600 ng/mL with six duplicated levels per concentration on the same day. The interbatch accuracy and precision were assessed over three days. The extraction recovery of photocyanine and IS was determined by calculating the ratio of the peak area of photocyanine and IS spiked in serum before extraction against postextraction spiked photocyanine and IS at the same concentration. The matrix effect was determined by calculating the matrix factor, which was obtained as the ratio of the analyte peak response in the presence of matrix ions to the analyte peak response in the absence of matrix ions by spiking analytes into blank serum extracts and blank water extracts. The stability of photocyanine was compared to the nominal level of photocyanine to determine whether photocyanine was stable in the experiments, including postpreparative stability test, freeze-thaw cycle test, and long-term stability test. If the calculated concentration of photocyanine was less than the nominal concentration by >15%, the analyte was considered to be unstable. Low, medium, and high serum QC samples were determined in six duplicated levels. The stability of the extracts was evaluated by putting them at room temperature for 24 h and then subjecting them to the analytical procedure. Photocyanine maintained at −80°C for 30 days was evaluated by comparing the postfreeze measured concentration with the initial concentration added to the sample. The freeze-thaw stability of the samples was assessed over three freeze-thaw cycles by thawing samples at room temperature, refreezing them for 24 h at −80°C and then analyzing them.

### 2.6. Application

Six patients with cancer were enrolled at the Cancer Center, Sun Yat-sen University. The patients were five males and one female, ranging in age from 37 to 69 years, who had been diagnosed with a primary or metastatic malignancy. All patients provided written informed consent prior to participation. The patients were infused (i.v. administration) with photocyanine (0.1 mg/kg) for 60 min. Blood samples were obtained at 0, 0.5, 1, 2, 4, 6, 8, 12, 24, 72, 120, and 168 h after administration and then placed on ice and kept away from light. The blood samples were centrifuged at 3000 rpm/min for 10 min, and the serum was stored at −80°C until the analysis was conducted. The present study was approved by the Human Subjects Review Committees of the University of Sun Yat-sen University Cancer Center and conducted according to the Declaration of Helsinki.

Noncompartmental pharmacokinetic parameters were calculated using WinNonlin (Version 5.0, PUMCH Clinical Pharmacology Research Center). The maximum serum concentration (*C*_max⁡_) and time to reach it (*T*_max⁡_) were determined directly from the data. The terminal-phase rate constant (*λ* *z*) was calculated as the negative slope of the log-linear terminal portion of the serum concentration-time curve using linear regression with at least four last concentration-time points. The terminal-phase half-life (*t*_1/2_) was calculated as 0.693/*λ* *z*. The area under the curve from time zero to the last observed time (AUC_0-*t*_) was calculated by the linear trapezoidal rule for ascending data points. The total area under the curve (AUC_0-∞_) was calculated as AUC_0-*t*_ + Ct/*λ* *z*, where Ct is the last measurable concentration. The apparent volume of distribution associated with the terminal phase (*Vz*) was calculated as* Vz* = CL/*λ* *z*, and the apparent total body clearance (CL) was calculated as CL = dose/AUC.

## 3. Results and Discussion

### 3.1. HPLC-MS/MS Condition Optimization

HPLC-MS/MS operation parameters were carefully optimized for the determination of photocyanine. Photocyanine is a typical sulfonate compound, which is usually ionized in negative mode. We tuned the mass spectrometer in both positive and negative ionization modes with ESI for photocyanine and found that both the signal intensity and ratio of signal to noise obtained in negative ionization mode were much greater than those in positive ionization mode, which was consistent with previous study on another sulfonate compound [19]. In the precursor ion full-scan spectra, the most abundant ions were protonated molecules with* m*/*z *ratios of 526.0 and 655.1 for photocyanine and IS, respectively. Parameters such as desolvation temperature, ESI source temperature, capillary voltage, and flow rate of desolvation gas and cone gas were all optimized to obtain the highest intensity of protonated analyte molecules. The product ion scan spectra showed high abundance fragment ions at* m*/*z *values of 146.0 and 591.8 for photocyanine and IS, respectively. Multiple reaction monitoring (MRM) using the precursor→product ion transition of* m*/*z *526.0→*m*/*z *146.0 and* m*/*z *655.1→*m*/*z *591.8 was used for the quantification of photocyanine and IS, respectively.

The efficiency of the chromatographic separation of photocyanine and IS was evaluated using tests with different chromatographic columns and mobile phases. Photocyanine is amphoteric and relatively hydrophobic. We found that photocyanine displayed a very strong retention on BDS or XDB C18 columns, and little retention on C8 or SCX columns, which resulted in broad peak or substantial carry over. These columns have been tested without success. It has been shown that good chromatographic profiles of photocyanine and IS are obtained using a Waters XBridge C18 column (50 mm × 4.6 mm, 5 *μ*m), with retention times of 2.33 and 2.59 min, respectively. The total analysis time per sample is 7 min, which is much shorter than that of 45 min in previous study [18]. We also optimized the column temperature by observing the chromatographic peak and resolution and found that XBridge C18 column displayed well column performance at 60°C. Optimization of the mobile phase is important for improving peak shape and detection sensitivity and for shortening the run time. We tested methanol, acetonitrile, and a mixture of the two as the organic modifier of the mobile phase, and we found that the peaks were more symmetric when methanol was used. Moreover, the chromatographic behavior of photocyanine subjected to mobile phases of different pH values was investigated, and we observed that deionized water (adjusted to pH 10.0 with aqueous ammonia) improved the peak shape and significantly increased the signal intensity of the analyte. In order to further optimize the chromatographic condition, the peak symmetry factor of photocyanine was calculated in different percentage of MPB at the elution period 1.3–4.0 min. Symmetry factor of a peak is calculated by dividing* W*_0.05_ by two-fold* f*, where* W*_0.05_ is the width of the peak at 5% height and* f* is the distance from the peak maximum to the leading edge of the peak, the distance being measured at a point 5% of the peak height from the baseline. As shown in Table 1, the optimal value was 95%. Finally, the optimized gradient elution with deionized water (adjusted to pH 10.0 with aqueous ammonia) and methanol at a flow rate of 0.6 mL/min was established in this study.

### 3.2. Sample Preparation Procedure

Sample preparation is important for the HPLC-MS/MS assay. Liquid-liquid extraction (LLE) and solid-phase extraction (SPE) are techniques often used in the preparation of biological samples due to their ability to improve assay sensitivity [20, 21]. SPE columns, including Strata, Strata-X, and Strata C18-E from Phenomenex (Torrence, CA, USA), OASIS WAX Cartridge, and Sep-pak C18 from Waters (Milford, MA, USA), were used for sample preparation in this study. However, photocyanine exhibited no elution due to its strong adsorption to the SPE columns. We also carried out LLE with ethyl acetate, n-butyl alcohol, and mixtures of these organic solvents with n-hexane; however, we obtained low recovery and reproducibility using this procedure. Because HPLC-MS/MS quantification is highly specific and sensitive, protein precipitation (PPT) from the sample preparations was tried in the present study. We found that PPT was not only simple and efficient but also applicable to pharmacokinetic studies in which only 100 *μ*L of serum was used to obtain bioanalytical results. In addition, we observed that the linearity of photocyanine concentration in human serum with DMF was significantly improved than that of without DMF, indicating that DMF is conductive to maximize the release of photocyanine in PPT by inhibiting the binding of the drug to serum proteins, but there is no related report so far about the mechanism of DMF in PPT or drug release. Finally, we added 200 *μ*L DMF to the sample, and the effect of DMF here is consistent to that in previous study [18].

### 3.3. Specificity

Specificity was determined by comparing the chromatograms of six different batches of blank human serum with the corresponding spiked serum. No interference from endogenous substances was observed at the respective retention times of photocyanine and IS (data not shown).

### 3.4. Linearity and LLOQ

**Figure 3:**
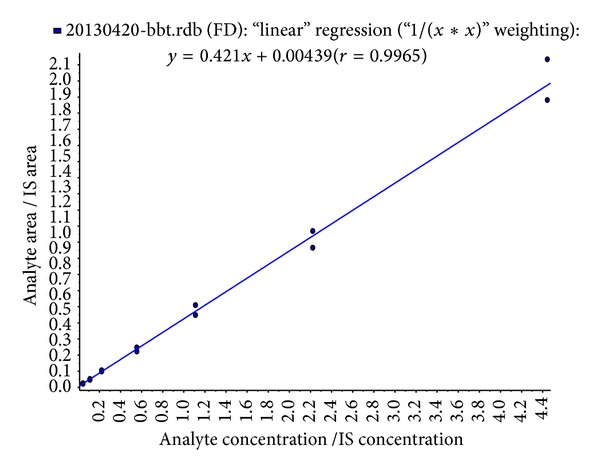
Linear calibration curve of photocyanine from the peak-area analyte/peak-area IS ratios versus concentrations in human serum using a weighting factor (1/*x* ^2^), varying linearly over the concentration range of 20–2000 ng/mL. The equation for the curve was *y* = 0.421*x* + 0.00439 (*r* = 0.9965).

The linear calibration curves were determined from the peak-area ratios (peak-area analyte/peak-area IS) versus concentration in human serum using a weighting factor (1/*x* ^2^), varying linearly over the concentration range tested (20–2000 ng/mL). As shown in Figure 3, the typical equation for the calibration curve for photocyanine was *y* = 0.421*x* + 0.00439 (*r* = 0.9965). The slopes of the equations were consistent with the calibration curves prepared on three separate days. The LLOQ in this study was 20 ng/mL for photocyanine, in which the S/N was >10, and the precision of repeat injections was 8.26%. Our method displayed a little higher sensitivity than the previous method, in which the LLOQ was 30 ng/mL [18].

### 3.5. Accuracy and Precision

The accuracy and precision of the method were determined by analyzing QC samples at three concentrations in six replicates. The intrabatch accuracy ranged from 101.98% to 107.54% at three concentrations, with precisions between 1.29% and 4.91%. The interbatch accuracy varied from 100.52% to 105.62%, with precisions ranging from 4.72% to 8.53% (Table 2). Thus, the present method has satisfactory accuracy, precision, and reproducibility.

### 3.6. Extraction Recovery and Matrix Effect

The extraction recoveries from QC samples at low, intermediate, and high concentrations ranged from 31.64% to 43.53% at three tested concentrations. We extracted the analyte from serum using protein precipitation and DMF in the present study, providing a simple and rapid method for the bioanalysis of photocyanine. In terms of matrix effects, the MF ranged from 61.51% to 77.03% at the three concentrations tested (Table 3), indicating that the coeluting substance only slightly influenced the ionization of the analyte.

### 3.7. Stability

The results from the stability tests are presented in Table 4, and the data demonstrate a good stability of photocyanine throughout the steps of the determination. The method is therefore applicable to routine analysis.

### 3.8. Analysis of Patient Samples

**Figure 4:**
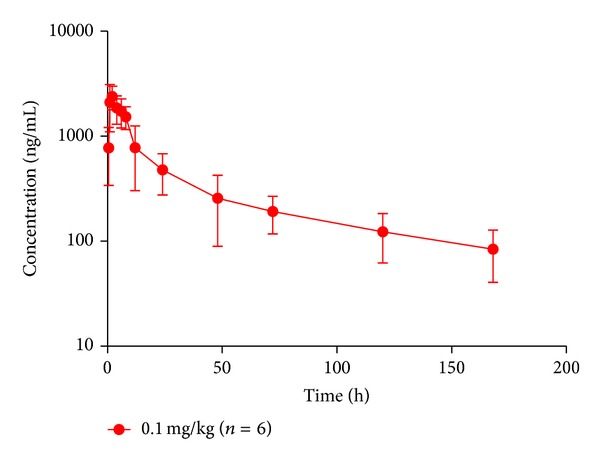
Mean serum concentration-time curve of photocyanine after 0.1 mg/kg single i.v. administration to cancer patients (*n* = 6).

The validated HPLC-MS/MS method described here was successfully applied to a pharmacokinetic study in 6 cancer patients following i.v. administration of 0.1 mg/kg photocyanine. A mean plasma concentration-time curve of a single dose of photocyanine is shown in Figure 4. This result revealed that our method was sufficiently sensitive to determine the photocyanine concentration in the serum of patients. The parameters of the pharmacokinetic analysis are shown in Table 5. The time of maximum plasma concentration (*T*_max⁡_) was 1.83 ± 0.41 h, the maximum plasma concentration (*C*_max⁡_) was 2465.3 ± 723.0 ng/mL, the half-life of drug elimination at the terminal phase (*t*_1/2_) was 74.62 ± 13.29 h, the area under the plasma concentration-time curve from 0 h to the time of the last detectable concentration (AUC_0-*t*_) was 53137.2 ± 20210.6 ng*·*mL^−1^ *·*h, the area under the plasma concentration-time curve from 0 h to infinity (AUC_0-∞_) was 62634.6 ± 25398.6 ng*·*mL^−1^ *·*h, the volume of distribution (*V*_*d*_) of photocyanine was 11.29 ± 4.12 L, the total clearance (CL) was 0.11 ± 0.04 L/h, and the mean residence time (MRT) was 40.16 ± 4.32 h.

## 4. Conclusion

A selective, sensitive, and rapid HPLC-MS/MS method for measuring photocyanine in human serum is described. In comparing this method with the analytical methods reported previously [14, 18], the present method proved superior with respect to higher simplicity of sample preparation, higher selectivity and sensitivity, and shorter chromatographic analysis time. The present description is the first to utilize the HPLC-MS/MS method for the pharmacokinetic study of photocyanine given by injection to cancer patients. 100 *μ*L human serum is sufficient for obtaining results in our pharmacokinetic study, indicating that the present method is applicable to human phase I clinical trials.

## Figures and Tables

**Table 1:** Peak symmetry factor of photocyanine in different percentage of mobile phase B.

MPB (%)	Symmetry factor (*n* = 5)	RSD (%)
95	1.052	4.67
90	1.103	9.00
80	1.198	7.45
70	1.354	7.67

**Table 2:** Precision and accuracy of photocyanine.

Concentration (ng/mL)	Intrabatch (*n* = 6)		Interbatch (*n* = 3)	
	Accuracy (%)	RSD (%)	Accuracy (%)	RSD (%)
60	103.72	4.91	105.62	4.72
1000	101.98	1.29	100.52	5.93
1600	107.54	3.31	101.18	8.53

**Table 3:** Extraction recovery and matrix effects of photocyanine.

Concentration (ng/mL)	Extraction recovery (%) (*n* = 5)	Matrix effect (%) (*n* = 6)
60	37.13	61.51
1000	43.53	71.24
1600	31.64	77.03

**Table 4:** Stability of photocyanine under different storage conditions.

Storage conditions	Concentration (ng/mL)	Accuracy (%) (*n* = 6)	RSD (%)
Freeze-thaw three cycles	60	107.08	6.09
	1000	101.20	4.14
	1600	93.92	1.76

−80°C for 30 days	60	95.14	10.01
	1000	89.56	1.85
	1600	85.31	3.26

Post-preparative (room temperature for 24 h)	60	102.52	12.12
	1000	96.16	3.55
	1600	102.15	2.00

**Table 5:** Noncompartmental pharmacokinetic parameters of photocyanine in cancer patients after a single i.v. dose of 0.1 mg/kg photocyanine (*n* = 6).

Parameters	Mean ± SD
*T*_max⁡_ (h)^a^	1.83 ± 0.41
*C*_max⁡_ (ng/mL)^b^	2465.3 ± 723.0
*t*_1/2_ (h)^c^	74.62 ± 13.29
AUC_0–*t*_^d^ (ng***·***mL^−1^ ***·***h)	53137.2 ± 20210.6
AUC_0–*∞*_^e^ (ng***·***mL^−1^ ***·***h)	62634.6 ± 25398.6
*V*_*d*_ (L)^f^	11.29 ± 4.12
CL (L/h)^g^	0.11 ± 0.04
MRT (h)^h^	40.16 ± 4.32

^a^ *T*_max⁡_: time to maximum concentration; ^b^ *C*_max⁡_: maximum plasma concentration; ^c^ *t*_1/2_: half-life of elimination; ^d^AUC_0–*t*_: area under the concentration-time curve from time zero to the last quantifiable measurement; ^e^AUC_0–*∞*_: area under the concentration-time curve extrapolated to infinity; ^f^ *V*_*d*_: volume of distribution; ^g^CL: total clearance; ^h^MRT: mean residence time.
